# The Preponderance of Evidence Indicates That the Eastern North American Monarch Population Has Declined in All Stages of Its Annual Cycle. Reply to Davis, A.K. Comment on “Oberhauser, K.S. Eastern North American Monarch Butterfly Conservation Needs and Opportunities: What the Science Tells Us. *Insects* 2026, *17*, 235”

**DOI:** 10.3390/insects17070713

**Published:** 2026-07-10

**Authors:** Karen S. Oberhauser, John Pleasants

**Affiliations:** 1 Department of Entomology, University of Wisconsin-Madison, Madison, WI 53706, USA; 2 Department of Ecology, Evolution, and Organismal Biology, Iowa State University, Ames, IA 50011, USA; jpleasan@iastate.edu

**Keywords:** monarch butterflies, milkweed reduction hypothesis, migration mortality hypothesis, insect conservation

## Abstract

Davis critiqued a recent review, “Eastern North American Monarch Butterfly Conservation Needs and Opportunities: What the Science Tells Us”. He argued that Eastern North American monarch numbers in the summer have not declined, despite dramatic declines in the area covered by this population in Mexico during the winter. These declines in winter numbers, he said, can be explained by increasing mortality during the fall migration and subsequent density-dependent increases the following spring. A strong impetus for these arguments appears to be Davis’s concern that recommendations by “certain circles” of the monarch research community could steer conservation funding toward a species that does not need our help. Here, we address these concerns, and note that there is no evidence that conservation funding focused on providing breeding habitat for monarchs detracts from conservation for other species in need of help, or from efforts to support migrating monarchs.

## Simple Summary

A recent review examined the causes of the decline in the size of the monarch butterfly population that overwinters in Mexico. The evidence indicates that herbicide use in agricultural fields has reduced the number of milkweeds in the summer breeding range, thus reducing the size of the population that migrates to Mexico. This conclusion has been challenged by Davis, who claims that the summer population has not declined and that decreasing migratory success is to blame. He faults the review for not considering studies that purport to show that the summer population has remained stable. He argues that conservation efforts to add milkweed to the landscape are unnecessary. Here, we examine the four studies he cites. Three of these are based on yearly surveys of adult monarchs or larvae. We cite several studies that show that yearly surveys conducted during the period when milkweeds were being eradicated from agricultural fields underestimated the size of the summer population and thus could not detect the population decline. Another study that found no genetic evidence of a population decline is open to interpretation. None of the four studies cited negates the preponderance of the evidence of milkweed decline leading to monarch decline.

This is a reply to the critique by Davis [1] of the recent review “Eastern North American Monarch Butterfly Conservation Needs and Opportunities: What the Science Tells Us” [2]. His main thesis is that summer monarch numbers have not declined and we should not be concerned about low winter numbers because the population can bounce back in the summer. Therefore, he argues, conservation efforts that focus on adding milkweed for summer breeding monarchs are misguided. Davis suggests that the reason for declining overwintering numbers is not declining milkweed numbers, the “milkweed reduction” hypothesis, but rather declining fall migration success, the “migration mortality” hypothesis. Here, we address his concerns and argue that these two hypotheses are not mutually exclusive.

From a commonsense perspective, Davis’s argument is fraught. Eastern North American overwintering and breeding monarchs are not separate populations; they are the same population. It makes no sense to talk about the “breeding population”. A decline in the size of the overwintering colonies without a concurrent decline in the number of breeding monarchs means they must somehow be making up the difference between their wintering sites in Mexico and their northern breeding grounds. The average area covered by monarchs in the first decade that colony area was measured (winters beginning 1993–2002) was 8.59 ha. During the most recent decade (winters beginning in 2016–2025), this average was 2.70 ha, over a three-fold difference (data from [3]). For summer numbers to consistently rebound every year in the most recent decade to the size of the earliest decade, population growth would have to average three times greater than in the past; an unlikely scenario.

The four lines of evidence Davis indicates for stable summer numbers, each of which we maintain are flawed, are as follows:

1. As evidence that summer numbers are not declining, Davis cites Crossley et al. [4], who analyzed summer (June–August) North American Butterfly Association counts at over 400 sites throughout North America, and an unpublished alternative analysis of these data. We do not have access to the alternative analysis, but as noted in the original review, Crossley et al. [4] document the highest monarch abundance in the Corn Belt of the US. Because this area contributes more to the whole population [5,6], trends in monarch numbers there have the largest impact on the whole population. It would have made more sense to assess trends in the southern US when the bulk of the population is there (in the spring and fall), but based on the published results, all we can say is that trends in relative summer abundance show a decline in areas where monarch numbers are highest (Figure 2a,b in [4]). Conversely, trends show an increase in areas where they were either assessed when monarchs are generally rare (the southern US in the summer) or where they are almost always rare (the Northwestern US and South-Central Canada).

As an explanation for the lack of correlation between winter and summer numbers, Crossley et al. [4] suggest density-dependent reproductive compensation and higher levels of migration mortality. Following a winter with low numbers, they suggest that monarchs experience high population growth, perhaps facilitated by reduced intraspecific competition. However, there is no evidence of increased summer survival as monarch numbers were declining. In fact, survival from the egg to fifth instar declined slightly from 1997 to 2014 [7]. Despite other claims [8], we lack convincing evidence that density-dependent factors reduce extinction risk in monarchs [9].

Crossley et al. [4] suggest that increased OE (*Ophryocystis elektroscirrha*) infection rates could be causing increased migration mortality. Majewska et al. [10] document an increase in parasite infection rates in the eastern migratory population and a relationship between infection rate and migration survival (migratory culling), but the infection rate increased from about 0.05% in 2000 to about 0.15% in 2019 (Figure 2c in [10]). It is difficult to imagine how this increase resulted in a three-fold decline in monarch winter numbers.

Even without these methodological and mechanistic concerns, Pleasants et al. [11,12] show that the study by Crossley et al. [4], and other studies that find a disconnect between summer counts and overwintering area, can be explained by the fact that those analyses include a time when the monarch breeding habitat was undergoing consequential change. Prior to the widespread adoption of crops genetically modified to withstand herbicide applications, most monarchs originated in agricultural fields, primarily corn and soybean fields where milkweed was plentiful [13]. The lack of a decline in summer surveys is the result of a sampling bias during the time this habitat was being lost (mid 1990s to about 2006) because agricultural fields were not being sampled. Consequently, counts made during that period underestimated the true size of the population in the summer. Counts made of migrating monarchs during that period, which include butterflies that come from all habitats and are thus a truer measure of summer numbers, are correlated with overwintering numbers [12]. When counts of adults and eggs were corrected for the proportion of habitat they represented, summer surveys were correlated with overwintering area [11]. This evidence supports the “milkweed reduction” hypothesis. After milkweed eradication from agricultural fields was complete, and this sampling basis no longer existed, both summer and fall surveys correlate with overwintering area [12]. The conclusions of studies that used summer counts during the eradication period, unless they correct for the bias in the type of habitat that was surveyed, are not credible.

2. Davis [1] also cites a recent genetic study [14] as evidence that monarch numbers are not declining. Boyle et al. [14] note uncertainty about the presence or absence of a recent bottleneck; five of the 20 runs of their genetic models predicted a bottleneck. To understand the uncertainty in these models, it is useful to think of the size of the eastern migratory monarch population. One hectare of overwintering butterflies constitutes approximately 20 million individuals [15]. At its lowest overwintering population level of 0.67 hectares in 2013, this still represents over 13 million individuals. Given the random mating that occurs at the end of the overwintering period, the lack of a recent loss of genetic diversity is not certain evidence that the population is stable. It may simply mean that the magnitude of the decline was insufficient to produce a detectable genetic signal. Boyle et al. [14] note this, giving three possible reasons for their findings: (1) there was not a decline, (2) a decline did occur, but it was too small to leave a signal, and (3) a decline occurred too recently to produce a genetic signal. The genetic methods used for this measurement of effective population size do not allow estimation of a minimum detectable event size, so it is impossible to assign more or less certainty to any of these possibilities. We note that the authors are more cautious about the interpretation of their findings than Davis: “We encourage restraint in the interpretation of these results and encourage parallel studies to test these ideas further…. We emphasize that our results do not directly bear on current efforts to support monarch butterfly conservation.” [14].

3. Davis’s third concern came from data from the Monarch Larva Monitoring Project (MLMP), which were analyzed by Pleasants et al. [12]. Davis created a graph from Supplementary Material in Pleasants et al. [12] showing MLMP egg densities from 1997 to 2021. He claimed that the data do not show evidence of population declines.

As noted above and in the original review as well as papers cited in the review, values from summer surveys conducted as milkweed was being eradicated during the period of herbicide-tolerant crop adoption do not reflect population accurately. MLMP eggs per milkweed stem data, if used properly, do corroborate a population decline. When eggs per stem were multiplied by the number of stems on the landscape, Pleasants et al. [11] showed a decline in total egg production as the number of stems declined and a corresponding decline in the overwintering population. The number of eggs per stem by itself does not present a picture of population size without accounting for the number of stems on the landscape.

4. In support of the “migration mortality” hypothesis, Davis cites Fordyce et al. [16], who had concerns about the statistical analysis used by Taylor et al. [17] to assess migration survival. Taylor et al. had two key conclusions. First, the size of the migratory (late summer) population, as measured by number of monarchs tagged, was strongly correlated with the size of the overwintering population, indicating that the size of the summer and the size of the winter populations are linked. Fordyce et al. [16] thought year should be included as a variable because of the decline in the overwintering population. But including year, which turns out to be a significant variable, acknowledges, as they point out, that both summer and winter numbers are declining. They also had concerns with the second conclusion, based on the percent of tags recovered in Mexico, that migration success did not show a decline. They pointed out that tags recovered could also be influenced by the motivation to search for tags and ability to find tags, both unknown sources of variation. Despite these unknowns, tag recovery rate does correlate slightly, but significantly, with NDVI (Normalized Difference Vegetation Index, which quantifies vegetation health), and overwintering area does correlate slightly, but significantly, with tag recovery rate [17]. These results suggest that migration success does have an effect, albeit small, on overwintering area, and that this effect could be driven by weather. We note that the Taylor et al.’s analysis [17] ended with data from 2014, and further examination of recovery rate with a longer-term data set could more definitively determine whether migration success is declining.

It is important to recognize that the trajectory of overwintering numbers shows two phases: a precipitous decline as milkweed was being eradicated from farm fields (1994–2006) and a shallower decline post-eradication [12]. Based on the regression relationships for these phases, the overwintering population went from 10.8 hectares in 1994 to 4.9 hectares in 2006 and from 3.4 hectares in 2007 to 2.1 hectares in 2025. Multiple lines of evidence point to the initial and more precipitous decline phase being due to loss of milkweed habitat [12,18,19]. The later, less precipitous decline may have several causes. There is continuing loss of milkweed habitat due to conversion of CRP land to farmland and human development [20,21]. The number of monarchs tagged, a measure of summer production, shows a decline consistent with this continuing loss of habitat [12]. But declining migration success, as suggested by evidence of declining roost size [22], could also be a factor. Reduced migration success could be related to climate change and its effect on migration itself as well as its effect on nectar resources that monarchs use along their migration journey. Increased disease levels could also be playing contributory roles. However, the main driver of overwintering numbers is summer production [12,19].

We note further that the “milkweed reduction” and “migration mortality” hypotheses as explanations for monarch population decline are not mutually exclusive. During the period of milkweed eradication, there is strong evidence for the “milkweed reduction” hypothesis being the major driver, and during the present, post-eradication, period, both milkweed reduction and increasing migration mortality could be contributing to the decline in overwintering numbers. As evidence for a combination of factors, Davis et al. [22] noted high synchrony in declining roost sizes across the flyway, but with support in the analytical models for declines increasing from north to south. We note that declines of the northern-most roosts ranged from 4% to 6% per year; these declines are due to lower numbers of breeding monarchs (and are incongruent with Davis’s claim that breeding numbers are not changing). The larger decline in the far south, up to about 10% along the Gulf Coast, is likely due to the combination of factors affecting breeding success and migration success.

Conclusions: Monarch Conservation

We disagree with the main point of Davis’s rebuttal, and argue that the preponderance of evidence documents a population that has declined during all stages of its annual cycle. However, we do recognize that signals from the breeding stage of this cycle are more difficult to interpret, as the original review pointed out. We would also like to comment on Davis’s suggestion that the review could “end up steering much-needed conservation funding towards a species that is not in need of help”. This statement is fraught from a conservation perspective. The precautionary principle, a generally accepted principle of environmental management [23,24], recognizes that decisions regarding environmental threats should be made in a way that gives the environment the benefit of any doubts about the risk. A lack of full scientific certainty regarding a risk should not be used as a basis for failure to protect against the risk. 

Additionally, providing habitat for monarchs, a charismatic species that attracts the attention of individuals who may not care a great deal about other butterflies and insects, will benefit many other species that are also in need of help. It will also help monarchs during their fall migration. There is no evidence that conservation funding that supports monarch breeding habitat will detract from conservation efforts for other insects or for monarchs during their fall migration. To support monarchs’ fall migration, Davis [22] recommends that people should provide “high-quality nectar sources and avoid planting non-native milkweeds that foster migration-taxing parasites”. Monarchs’ southward migration begins at the northern reaches of their breeding range and essentially retraces the entire breeding range; this means that the breeding and migratory ranges are not different. High quality breeding habitat includes high quality nectar plants that support both breeding and migratory monarchs, and native milkweeds, and thus addresses both of Davis’s recommendations. As noted by Oberhauser [2], there is wide consensus on the risks associated with the presence of non-native milkweeds during the fall migration.

In summary, as members of the “circle of the monarch research community” that advocates for creating more breeding habitat across the monarch range, we have weighed evidence from many sources and argue that the preponderance of evidence documents a population that has declined since we began measuring it. As pointed out in the review in question [2], it has stabilized or is at least declining more slowly in recent years, but at a level that is at significant risk of reaching dangerously low levels in the future and is vulnerable to mass mortality events in the overwintering area. This requires the addition of more habitat to achieve a resilient population.
